# Physiotherapeutic Scoliosis-Specific Exercise Methodologies Used for Conservative Treatment of Adolescent Idiopathic Scoliosis, and Their Effectiveness: An Extended Literature Review of Current Research and Practice

**DOI:** 10.3390/ijerph19159240

**Published:** 2022-07-28

**Authors:** Vaiva Seleviciene, Aiste Cesnaviciute, Birute Strukcinskiene, Ludmiła Marcinowicz, Neringa Strazdiene, Agnieszka Genowska

**Affiliations:** 1 Faculty of Health Care, Vilnius University of Applied Sciences, LT-08303 Vilnius, Lithuania; 2 Physical Medicine and Rehabilitation Centre, Vilnius Children’s Hospital, Vilnius University Hospital Santaros Klinikos, LT-08661 Vilnius, Lithuania; 3 Department of Physical Medicine and Rehabilitation, Mykolas Marcinkevicius Hospital, LT-03215 Vilnius, Lithuania; cesnaviciute3@gmail.com; 4 Faculty of Health Sciences, Klaipeda University, LT-92294 Klaipeda, Lithuania; birute.strukcinskiene@ku.lt; 5 Department of Obstetrics, Gynecology and Maternity Care, Medical University of Bialystok, 15-295 Bialystok, Poland; ludmila.marcinowicz@umb.edu.pl; 6 Faculty of Social Sciences and Humanities, Klaipeda University, LT-92294 Klaipeda, Lithuania; n.strazdiene@gmail.com; 7 Department of Public Health, Medical University of Bialystok, 15-295 Bialystok, Poland

**Keywords:** physiotherapeutic scoliosis-specific exercises (PSSE), adolescent idiopathic scoliosis (AIS), conservative treatment, methods, Schroth, SEAS, BSPTS, DoboMed, FITS, FED, Lyon, Side Shift

## Abstract

Due to the multifactorial etiology of scoliosis, a comprehensive treatment plan is essential for conservative management. Physiotherapeutic scoliosis-specific exercise (PSSE) methods have lately gained popularity for the conservative treatment of scoliosis. The aim of this study was to analyze the PSSE methodologies used for conservative treatment of adolescent idiopathic scoliosis (AIS), as well as their effectiveness. The study was based on an extended literature search conducted in the PubMed, Google Scholar, PEDro, eLABA, and BioMed Central databases. A total of 123 articles were selected for this study (including articles overviewed in systematic reviews and meta-analyses) after applying the inclusion criteria. The study revealed that inappropriate management of AIS could result in serious health problems. Conservative interventions that aid in stabilizing spine curvature and improving esthetics are preferred for scoliosis treatment. Bracing has traditionally been the mainstay of treatment, but growing evidence suggests that PSSE physiotherapy allows effective management of idiopathic adolescent scoliosis. Currently, there are the following PSSE physiotherapy schools in Europe: Schroth, SEAS, BSPTS, FED, FITS, Lyon, Side Shift, and DoboMed. The methodologies of these schools are similar, in that they focus on applying corrective exercises in three planes, developing stability and balance, breathing exercises, and posture awareness. Although high-quality research supporting the effectiveness of PSSE physiotherapy in the treatment of AIS is lacking, existing evidence indicates that PSSE physiotherapy helps to stabilize spinal deformity and improve patients’ quality of life. Among the abovementioned methodologies, Schroth is the most widely studied and has been proven to be effective. However, both SEAS and BSPTS effectively stabilize and even reduce the Cobb angle of scoliosis. Data supporting the validity of other methodologies are very limited. Only the Schroth method significantly reduces the angle of trunk rotation, while both SEAS and Schroth methods greatly improve the quality of life indicators. In any case, the available evidence is insufficient to confirm the advantage of one specific physiotherapy technique over others.

## 1. Introduction

Scoliosis refers to a complex deformity of the spine in three planes. It is diagnosed by measuring the angle of curvature of the spine (a Cobb angle of at least 10°) in the frontal plane and the axial rotation in the horizontal plane, as well as being characterized by spinal deformities in the sagittal plane [1,2]. Adolescent idiopathic scoliosis (AIS) is a three-dimensional spine deformity. It is a structural, lateral, rotated curvature of the spine that arises in otherwise healthy children at or around puberty [3,4]. Although scoliosis can be diagnosed at any age, the majority of cases are detected during adolescence between 10 and 18 years [2,3,4,5]. Scoliosis is the most common pediatric deformity of the spine [6,7]. The International Society on Scoliosis Orthopaedic and Rehabilitation Treatment (SOSORT) has estimated that the global incidence of adolescent idiopathic scoliosis (AIS) in pediatric population ranges from 0.93% to 12% [8].

Adolescent idiopathic scoliosis is a multifactorial disease with intrinsic and extrinsic alterations. The key points of the etiology and pathogenesis of AIS were found to be related with changes in almost every field from genetic to environmental factors [4]. However, about 80% of scoliosis cases present with idiopathic scoliosis of unknown etiology [8]. Scoliosis is called idiopathic when no other underlying disease can be identified [9].

Inappropriate treatment of AIS can result in severe deformities of the trunk, back, and chest, disturbing the functional biomechanics of the body, limiting lung volume, and reducing physical capacity, work capacity, and quality of life [8]. Although scoliosis has long been thought to be a harmless condition, evidence indicates that people with scoliosis are more prone to experience back pain [10,11]. Scoliosis causes disability of varying severity and poses a high healthcare burden, particularly when patients require extensive surgical treatment [12,13]. Due to the complexity of the procedure, the surgical treatment for scoliosis involves a relatively high cost and risk of complications. Because the risks associated with mild and moderate scoliosis are very high, conservative treatment is preferred to stabilize the deformity and improve the quality of life.

As idiopathic scoliosis is characterized by a complex pathomechanism, a comprehensive treatment plan is essential for its conservative management. One of the best approaches for scoliosis management is to use physiotherapeutic scoliosis-specific exercises (PSSE). The SOSORT uses the term physiotherapeutic scoliosis-specific exercises (PSSE) for all schools and methods approved by the organization. Every method and school incorporate the principles of the SOSORT guidelines and share a common mission. A PSSE physiotherapy methodology must be based on scientific evidence and customized for each patient [14,15]. The SOSORT consensus attributes to the “3D self-correction” first place in the ranking of important elements to be included in the exercises [16,17]. Self-correction can be defined as the search for the best possible alignment the patient can achieve in the three spatial planes [18].

The SOSORT emphasizes that PSSE physiotherapy used for the treatment of AIS differs from nonspecific physiotherapy in that it aims at three-dimensional self-correction of posture, stabilization of corrected posture, patient education, and the integration of corrective positions into daily activities [8]. The frequency of PSSE physiotherapy varies from 2 to 7 days per week, depending on the complexity of the technique used, the need of the patients, and their ability to follow the prescribed program. Long-term outpatient programs typically occur two–four times a week if the patient is ready to cooperate [15]. Typically, PSSE physiotherapy is only performed by professionally trained instructors, as physical exercises are chosen based on the type and severity of scoliosis in each patient, and the nature of the exercises themselves depends on the methodology applied [8].

The most well-known PSSE physiotherapy schools operating under the SOSORT are as follows [15]:

- Schroth, Germany;
- Lyon, France;
- SEAS (Scientific Exercise Approach to Scoliosis), Italy;
- BSPTS (Barcelona Scoliosis Physical Therapy School), Spain;
- Side Shift, UK;
- DoboMed, Poland;
- FITS (Functional Individual Therapy of Scoliosis), Poland; and
- FED, Spain.

Each PSSE physiotherapy methodology has been developed and applied in different European countries (see Table 1). Several other methodologies are also commonly used in different continents, but their principles are similar to those used in Europe.

Despite the fact that each of these schools has developed a separate methodology for the treatment of scoliosis and has been applying it for decades, the scientific validity of both PSSE physiotherapy and nonspecific physiotherapy in the treatment of AIS remains in question [26]. According to the Cochrane Systematic Review, scientific works published prior to 2012 do not provide strong evidence supporting the effectiveness of physiotherapy in the treatment of AIS [27,28], but subsequent publications have confirmed that physiotherapy can help reduce the Cobb angle of scoliosis and improve the quality of life [29,30]. However, due to the complexity of PSSE physiotherapy and research limitations, it is difficult to draw conclusions about the effectiveness of this therapy, and many authors have emphasized that high-quality research on the effectiveness of physiotherapy in scoliosis treatment is lacking [31,32,33,34]. Therefore, this review aimed to analyze the PSSE methodologies used for conservative treatment of adolescent idiopathic scoliosis (AIS), as well as their effectiveness.

## 2. Materials and Methods

This paper provides an extended overview of PSSE interventions. We systematically reviewed relevant PSSE methodologies, assessed the available evidence on their effectiveness, and oversaw possible directions for future research on PSSE physiotherapies. In addition, awareness of the best and most recent evidence allows for an optimal clinical decision [35]. Numerous studies have been completed on this topic, but no study has synthesized this information in the form of an extensive literature review. 

This study integrated previous research conducted on the PSSE treatment of AIS, published from 2010 through May 2021. The study was based on an extended literature search performed in the PubMed, Google Scholar, PEDro, eLABA, and BioMed Central databases. The keywords used for the search were scoliosis, adolescent idiopathic scoliosis, Schroth, FITS, DoboMed, SEAS, Side Shift, and FED, as well as various combinations of these words. The publications were selected in accordance with the following inclusion criteria: the object of research should be related to adolescent idiopathic scoliosis; studies had to be investigating PSSE methods (Schroth, FITS, DoboMed, Side Shift, BSPTS, Lyon, SEAS, FED, or others); meta-analyses and systematic reviews had to focus on the effectiveness of PSSE on AIS. The exclusion criteria applied for the selection of publications were the following: age of the participants was <10 and >18 years; cases where PSSE was used during the postoperative period; cases where the use of bracing or other specific conservative treatment interventions without PSSE were employed; cases where PSSE was used in combination with other physical exercise interventions; studies where the sample size was <10.

A total of 123 articles were reviewed in this work (including articles overviewed in systematic reviews and meta-analyses). The effectiveness of PSSE methodologies was analyzed in 13 controlled clinical trials published after 2015, and 10 systematic reviews and 7 meta-analyses published after 2010.

In our work, we used and applied such concepts as *effectiveness, efficacy, and effect. Effectiveness* is the ability of an intervention to have a meaningful *effect* on patients in normal clinical conditions. *Effectiveness* relates to how well treatment works in practice, as opposed to *efficacy*, which is the capacity of a given intervention under ideal or controlled conditions. *Effectiveness* is doing “the right” things, for example setting the correct targets to achieve an overall goal (the *effect*). It is the extent to which planned outcomes, goals, or objectives are achieved as a result of an activity, intervention, or initiative intended to achieve the desired *effect*, under ordinary circumstances [36].

## 3. Results

### 3.1. Effects of PSSE Physiotherapy Methods in the Treatment of AIS

The effectiveness of PSSE physiotherapy can be assessed based on various factors. The main indicators, as mentioned in many articles, are the change in the Cobb angle and the angle of trunk rotation (ATR). The secondary factors considered for the assessment of effectiveness are the improvement in quality of life, functionality, disability, and pain, aesthetic improvement in terms of deformity, delay of progression (postponing bracing or surgery), and others.

In the last decade, Schroth and SEAS methodologies have been used most frequently to investigate the effectiveness of PSSE physiotherapy, whereas other methodologies have not been extensively studied and their validity is based on previous research. An overview of randomized and nonrandomized controlled clinical trials over the past 5 years is presented in Table 2 and Table 3. Eight publications [37,38,39,40,41,42,43,44] examined the effectiveness of Schroth, four [39,45,46,47] examined SEAS, one [48] examined BSPTS, and one [25] examined FED using the FITS methodology in the control group. In other studies, observation, traditional physiotherapy, Pilates exercises, core stabilization exercises, or corset wear without physical exercise intervention were used in the control groups. Two studies compared the effects of different methodologies [25,39]. Two meta-analyses evaluated the effectiveness and effects of only Schroth [32,48]. One systematic review assessed the effectiveness of the FED methodology [49]. Other PSSE physiotherapy methodologies have not been studied in systematic reviews or meta-analyses.

#### 3.1.1. Effects of PSSE Physiotherapy on Cobb Angle

Analysis of the latest scientific publications revealed much data supporting that purposeful physical exercise can stabilize or even reduce the size of a scoliotic spinal deformity. A systematic analysis conducted by Anwer et al. in 2015 showed the effects of exercise on the spinal deformities of AIS. Four of the six studies included in this systematic analysis [43,50,51,52] analyzed changes in the measured Cobb angle, and two of them were randomized controlled clinical studies [43,50]. Of the four studies, three were rated above 6 on the PEDro scale [43,50,51] and revealed a statistically significant change in the groups that received physical exercise intervention. Medium-quality evidence showed that exercise interventions reduced the Cobb angle, and the degree of heterogeneity was determined to be average (*I*^2^ = 30.53%) [30]. Two of the studies used exercises based on the Schroth methodology for the intervention group [43,51], and two studies used an exercise program based on the SEAS methodology [50,52]. Statistically significant changes were observed only when PSSE physiotherapy was used.

In 2018, Farooqui et al. conducted a meta-analysis of 17 randomized controlled clinical trials to evaluate the effects of different exercise-based therapeutic interventions in conservative scoliosis management. Two of the analyzed articles were also reviewed by Anwer et al. [43,50]. Statistical analysis showed no significant evidence that exercise-based interventions help to reduce the degree of pathological spinal curvature in patients with AIS. The authors concluded that most of the studies included in the meta-analysis provided low-quality evidence; the study protocols were examined on a small sample of subjects, and there were differences in control groups and control strategies [30]. Among the studies included in the meta-analysis, three used the Schroth intervention [44,53,54], one used SEAS [47], and one used Side Shift [55]. The remaining studies used other exercise interventions.

**Table 2:** Characteristics of controlled clinical trials performed in 2016–2021.

Study	Sample	Age	Cobb Angle at the Beginning of the Study	Protocol of Intervention	
				Experimental Group	Control Group
Kocaman, 2021 [38]	28	10–18	10–26°	**Schroth:** 90 min, three times per week	**Trunk muscle endurance exercises:** 90 min, three times per week
Shah, 2020 [39]	30	10–18	20–45°	**Schroth:** Five times per week	**SEAS:** Five times per week
Trzcińska, 2020 [25]	60	11–15	30–60°	**Bracing + FED:** 15 min electrostimulation + 15 min heating procedure + 30 min FED + 20–30 min asymmetric exercise. 15 h, twice per day, three weeks	**Bracing + FITS:** 15 h, twice per day, three weeks
Negrini, 2019 [45]	293	10–14	11–20°	**1 group SEAS:** Four sessions with physiotherapist per year, 15 h every day + home program 90 min per week **2 group nonspecific physical therapy:** 60–90 min, twice per week	**Observation** or <15 min per session and 45 min per week
Yagci, 2019 [46]	30	12–16	20–45°	**Bracing + SEAS:** Sessions led by a physiotherapist: duration—40 min, once per week. Home program: 20 min per day	**Bracing + trunk muscle strength training:** Duration is the same as in the experimental group
Zapata, 2019 [56]	49	10–17	12–20°	**BSPTS:** In total, at least 8 h with instructor per 6 months + home program: 15 min per day, three times per week	**Observation**
Zheng, 2018 [47]	53	10–14	21–36°	**SEAS:** Training in a clinic led by a physiotherapist—40 min, once per week. Home program: 10–15 min per day	**Bracing:** 23 h per day
Kwan, 2017 [41]	48	10–14	25–40°	**Bracing+ Schroth:** An 8 week outpatient program + home program + revisit every 2 months + bracing 18 h per day	**Bracing:** 18 h per day
Strukčinskaitė, 2017 [37]	50	9–17	10–45°	**Schroth:** 10 procedures—30 min per procedure	**Physiotherapy:** 10 procedures—30 min per procedure
Kim, 2016 [40]	15	13–23	16–40°	**Schroth + respiratory muscle training:** 15 min respiratory exercises + 40 min Schroth exercises three times per week	**Only Schroth exercises:** 1 h session, three times per week
Kim, 2016 [42]	24	14–17	10–27°	**Schroth:** 60 min, three times per week, 12 weeks in total	**Pilates exercises:** 60 min, three times per week, 12 weeks in total
Kuru, 2016 [43]	45	11–14	20–50°	**1 group: Schroth training with rotational breathing led by a physiotherapist:** 15 h per day, three times per week, totally 6 weeks + home program **2 group: An independent Schroth program at home without the supervision of a physiotherapist + rotational breathing:** 15 h per day, three times per week	**Observation only**
Schreiber, 2016 [45]	50	13–14	10–45°	**Schroth:** A 5 × 60 min outpatient rehabilitation sessions for the first 2 weeks, and then 60 min once a week + 30–45 min of daily exercise at home for a total of 6 months	**Routine supervision:** Observation or bracing, if the patient meets the criteria for bracing

**Table 3:** Results of controlled clinical trials performed in 2016–2021.

Study	Duration of the Study	Execution of the Program	Evaluated Indicators				Results
			Cobb	Trunk Asymmetry	Quality of Life	Other	
Kocaman, 2021 [38]	10 weeks	100%	✓	✓ ATR, WRVAS	✓ SRS-22 (initial average 3.5)	✓ Spinal mobility	1. Schroth’s method is superior to trunk muscle stabilization exercises in reducing Cobb angle (*p* < 0.001) and ATR (*p* < 0.001) in cases of thoracic scoliosis, but not lumbar scoliosis (*p* > 0.05). 2. Schroth showed significant advantages in the assessment of quality of life (EG: 4.56, CG: 4.3, *p* < 0.05), as well as in the assessment of spinal mobility and asymmetry according to WRVAS.
Shah, 2020 [39]	7 weeks	Not indicated	✓	-	-	-	1. Significant change in Cobb was noted in the Schroth group (mean Cobb: 31.2° ± 5.2° before intervention; 27.4° ± 5.17° after intervention). There was also a significant change in Cobb in the SEAS group (31.33° ± 5.26° before intervention and 29.4° ± 5.9° after intervention). 2. The change in Cobb in the Schroth group was significantly greater than in the SEAS group (*p* < 0.001).
Trzcińska, 2020 [25]	3 weeks	Not indicated	✓	-	-	-	1. Significant change in Cobb was found within and between groups—FED (change in Cobb): –13.39° ± 8.66, *p* < 0.001; FITS (change in Cobb): –4.87° ± 10.94, *p* < 0.001; between groups: *p* < 0.01.
Negrini, 2019 [45]	2.1 ± 1.3 years	EG1: 81% EG2: 90% CG: 79%	✓	✓ ATR, hump height, TRACE	-	✓ Need for bracing	1. Significant change in Cobb was noted in the SEAS group (Cobb difference: 1.70° ± 7.24°), nonspecific physiotherapy group (Cobb difference: 2.08° ± 7.36°), and control group (Cobb difference: 2.20° ± 6.25°); *p* < 0.01. 2. The ATR was insignificant in the groups, and the height of the hump statistically significantly decreased only in the SEAS group (*p* < 0.05). 3. A statistically significant change in aesthetics was found in the SEAS group (change: –1.41 ± 2.22, *p* < 0.01) and nonspecific physiotherapy group (change: –0.91 ± 2.37, *p* < 0.01). 4. Both SEAS and nonspecific physiotherapy significantly reduced the need for bracing (SEAS: *F*-test 0.004, nonspecific physiotherapy: *p* = 0.04), but the advantage of SEAS over nonspecific physiotherapy was insignificant.
Yagci, 2019 [46]	4 months	EG: 64% CG: 62%	✓	✓ ATR, POTSI, WRVAS	✓ SRS-22 (initial average: 4.0)	-	1. Cobb difference: −5.3° ± 2.2° versus −4.8° ± 2.6° in the thoracic curves; –4.1° ± 2.5° versus –3.5° ± 3.0° in the lumbar curve; between groups: *p* > 0.05 (insignificant difference). 2. No difference in ATR, POTSI, and WRVAS was found between groups. 3. The pain rate improved only in the control group (4.7 versus 4.3).
Zapata, 2019 [56]	1 year	EG: 6% CG: 61%	✓	✓ SAQ	✓ SRS-22 (initial average: 4.4)	-	1. After 6 months, the difference in Cobb angle between groups was insignificant. After 1 year, the difference in Cobb between groups was significant (16.3° versus 21.6°, *p* = 0.04). 2. The change in the SAQ data within and between groups was insignificant. 3. The change in the SRS-22 data within and between groups was insignificant.
Zheng, 2018 [47]	1 year	EG: 59 ± 0.2% CG: 58 ± 0.27%	✓	✓ Shoulder level, TAPS, ATI	✓ SRS-22 (initial average: 4.2)	-	1. The Cobb angle decreased more in the bracing group (5.58° ± 6.37° versus 2.24° ± 3.19°). 2. Shoulder balance was improved in the bracing group. 3. The scores concerning quality of life, especially function (*p* ˂ 0.001), mental health (*p* ˂ 0.001), and total score (*p* ˂ 0.001), were higher in the exercise group.
Kwan, 2017 [41]	18.1 ± 6.2 months	EG: 77% CG: 79%	✓	✓ ATR	✓ SRS-22 (initial average: 4.2)	-	1. In the EG group, Cobb angle decreased in 17%, stabilized in 62%, and increased in 21% of patients; in the CG group, Cobb angle decreased in 4%, stabilized in 46%, and increased in 50% of patients. 2. No difference in ATR was found between groups. 3. SRS-22 data were more favorable for EG (functional activity domain 4.8 versus 4.6).
Strukčinskaitė, 2017 [37]	2 weeks	Not indicated	-	✓ ATR, DIERS 3D parameters	✓ SRS-22 (initial average: 4)	✓ Trunk static endurance, spinal mobility	1. Significant change in ATR was found in the experimental group (from 6.04° to 5.32°, *p* < 0.05). However, no significant difference was found between groups. 2. The SRS-22 data were slightly more favorable for EG (only 4.42 ± 0.66 versus 4.18 ± 0.92 in the treatment satisfaction domain). 3. When comparing DIERS 3D parameters, no significant difference was found between groups (*p* > 0.05). 4. Trunk static endurance and spinal mobility data in EG and in CG groups improved significantly after rehabilitation (*p* < 0.05); among the groups, the EG group showed better results (*p* < 0.05).
Kim, 2016 [40]	8 weeks	Not indicated	✓	-	-	✓ Respiratory function	1. Difference in Cobb angle change was found between groups—EG: 4.26° ± 1.36°, CG: 2.69° ± 1.11°, *p* < 0.05. 2. A significant change in peak expiratory flow was found between groups (EG: −1.30 ± 0.87, CG: −0.17 ± 0.68, *p* < 0.05).
Kim, 2016 [42]	3 months	Not indicated	✓	-	-	✓ Weight distribution	1. A significant (*p* < 0.05) change in Cobb angle was observed: Schroth group—before intervention: 23.6° ± 1.5°, after intervention: 12.0° ± 4.7°; Pilates group—before intervention: 24.0° ± 2.6°, after intervention: 16.0° ± 6.9°. 2. No changes in weight distribution were observed in CG, but a significant change (*p* < 0.05) was recorded in EG.
Kuru, 2016 [43]	24 weeks	Not indicated	✓	✓ ATR, hump height, trunk asymmetry	✓ SRS-22 (initial average: 3.9)	-	1. Positive changes in Cobb angle (−2.53°, *p* < 0.001), ATR (−4.23°, *p* < 0.001), hump height (−68.66 cm, *p* < 0.01), and lumbar asymmetry (*p* < 0.01) were observed in the physiotherapist-guided exercise group compared with the home exercise program group and the control group at week 24. 2. Differences in quality of life were not observed between groups (4.4 versus 3.9 versus 4.1).
Schreiber, 2016 [44]	6 months	With physiotherapist: 76% Home program: 73%	✓	-	-	-	1. A change in Cobb angle was found between groups (Cobb difference—EG: −3.5°, CG: +2.3°, *p* < 0.01). Cobb angle decreased significantly in the main curvature of scoliosis, and the size of all summed deformation angles decreased by 0.4° (*p* < 0.05 between groups).

Abbreviations are as follows: ATI—angle of trunk inclination; ATR—angle of trunk rotation; CG—control group; DIERS 3D—DIERS spinal and posture diagnostic system; EG—experimental group; POTSI—Posterior Trunk Symmetry Index; SAQ—Spinal Appearance Questionnaire; SRS-22—Quality of Life questionnaire of the Scoliosis Research Society; TAPS—Trunk Appearance Perception Scale; TRACE—Trunk Aesthetic Clinical Evaluation; WRVAS—Walter Reed Visual Assessment Scale.

In 2019, Thompson and co-authors narrowed the range of physical exercise interventions. They performed a systematic literature review and meta-analysis examining the effectiveness of PSSE physiotherapy compared to other nonsurgical interventions, such as traditional physiotherapy. Six controlled clinical trials were included in the meta-analysis [43,44,50,54,57,58]. Three of them [44,50,54] were also included in the review of Anwer et al. and Farooqi et al. Statistical analysis revealed that the group that underwent PSSE physiotherapy showed a statistically significant decrease in the thoracic Cobb angle by 6.9° (95% CI [–9.04; –4.70]), and a decrease in the lumbar Cobb angle by 7.2° (95% CI [–9.98; –4.48]) and the main spinal curve by 5.0° (95% CI [–8.95; –1.05]). However, four of the six randomized clinical trials were characterized by a high risk of bias, and the positive studies had followed an overall low-quality protocol [59]. The studies that were not included in the above analyses did not apply a specific methodology of the physiotherapy school.

In the same year, Day and co-authors performed a meta-analysis of eight randomized clinical trials [43,44,50,51,52,60,61,62]. Two of the trials were excluded from the analysis due to data insufficiency [60,61]. The remaining six trials were examined to determine the effect of PSSE on pathological spinal curvatures in scoliosis in comparison to no intervention and to nonspecific physiotherapy. One study that was not previously mentioned [63] used the SEAS methodology. Reliable results could not be obtained due to the high heterogeneity of the studies (PSSE physiotherapy versus no intervention: *I*^2^ = 84.2%; PSSE physiotherapy versus nonspecific physiotherapy: *I*^2^ = 96.75%) [34]. Similarly, Burger and co-authors, for the same reason, failed to perform a meta-analysis when investigating the effect of PSSE physiotherapy on spinal pathological curves in AIS [32]. Park et al. performed a meta-analysis of 15 publications on the effectiveness of the Schroth methodology [48]. The authors also observed high research heterogeneity, so they applied a random analysis model to the meta-analysis. Their analysis proved that the Schroth method is most effective in the treatment of mild (10–30° Cobb) scoliosis, while a positive effect can also be expected in more severe cases. A higher effect was observed when the Schroth therapy was continued for more than 1 month [48]. Burger and co-authors revealed that Schroth exercises significantly reduced the Cobb angle and improved the quality of life in adolescents with idiopathic scoliosis, and a short-term application of the methodology (exercises at 12 weeks and at 24 weeks) led to significant regression of the Cobb angle [32]. The authors attributed this finding to the supervision of a higher number of procedures by a physiotherapist at the beginning of treatment, which may lead to more intense and purposeful physical activity. Given that the average duration of scoliosis treatment is about 2–3 years, short-term studies may not provide accurate results, and only data from long-term studies on treatment effect should be considered.

Among controlled clinical trials conducted over the past 5 years, the effectiveness of treatment was monitored for more than 6 months in six trials (Table 3). Two studies on Schroth exercises investigated the effectiveness of treatment for 6 [43,44], and 18.1 months [41], respectively. Studies analyzing the effectiveness of the SEAS methodology were conducted for a period of 25 [45] and 12 months [47], and a study on the use of the BSPTS methodology [56] for 1 year was also analyzed.

A study by Zheng and co-authors [47] indicated that the SEAS methodology had no significant advantage over bracing for 23 h per day. A higher decrease in Cobb angle was noted in the bracing group (SEAS: 5.58° ± 6.37°, bracing: 2.24° ± 3.19°), which led to speculation concerning the effectiveness of the SEAS methodology. Nevertheless, in a study of twice the duration and with a significantly larger sample (*n* = 53 versus *n* = 293), Negrini and co-authors found that the SEAS methodology was better in preventing the progression of Cobb angle compared to traditional physiotherapy as well as observation (control). A significant change in the Cobb angle was observed in the SEAS group (Cobb difference: 1.70° ± 7.24°) in comparison to the nonspecific physiotherapy group (Cobb difference: 2.08° ± 7.36°) and control group (Cobb difference: 2.20° ± 6.25°) (*p* < 0.01) [45]. In all the studied groups, the change in Cobb angle was positive, so scoliosis progressed regardless of the intervention applied, with the lowest progression recorded in the SEAS group.

Analysis of data from two long-term (6 month) studies on the effectiveness of the Schroth methodology showed a reduction in Cobb angle in both studies [43,44]. In the study of Kuru and co-authors, the authors observed a reduction in the Cobb angle by 2.53° in the group that underwent Schroth procedures performed by a physiotherapist (*p* < 0.001). On the other hand, no significant changes were observed in the Schroth home program group and control observational group. Schreiber and co-authors also recorded a significant change in the Cobb angle of the main spinal curve in the Schroth group (−3.5°, control group: + 2.3°, *p* < 0.01) and determined the between-group difference in the square root of the Sum of Curves at –0.40°, (*p* = 0.046). Both studies showed a significant reduction in the angle of scoliosis but, due to small sample sizes (*n* = 45 and *n* = 50, respectively), the reliability of the data remains in question. In a longer-term study by Kwan and co-authors (18.1 months), the Cobb angle decreased in 17%, stabilized in 62%, and increased in 21% of patients in the Schroth group, whereas in the control group it decreased in 4%, stabilized in 46%, and increased in 50% [41]. This suggests that the Schroth methodology, like SEAS, is an effective intervention to stop the progression of scoliosis.

In 2020, Shah and co-authors [39] performed a study comparing Schroth and SEAS methodologies. The obtained data revealed that both methodologies were effective in reducing the Cobb angle (Schroth group—before intervention: 31.2° ± 5.2°, after intervention: 27.4° ± 5.17°; SEAS group—before intervention: 31.33° ± 5.26°, after intervention: 29.4° ± 5.9°). A statistically significant advantage of Schroth PSSE was found among the groups (*p* < 0.001). However, the sample size of the study was small (*n* = 30), and the study lasted only 7 weeks. As indicated by Burger et al. [32], the impact of the Schroth methodology observed in shorter-term studies is often stronger and, therefore, there is a need to perform detailed studies of the used methodologies. 

Studies have shown that the BSPTS methodology is also effective in stabilizing the progression of scoliosis. In a 1 year study by Zapata and co-authors, the BSPTS group showed effective stabilization of the Cobb angle compared to the observation group [56]. However, after 6 months, the difference in Cobb angle between groups was insignificant, which suggests that BSPTS is only effective if this methodology is followed for more than 6 months. The sample size of the study was *n* = 49, and the participants had only mild scoliosis (Risser score = 0). In the experimental group, scoliosis progressed by 16%, while in the control group it progressed by 50%. This indicates that BSPTS has the potential to stabilize scoliosis deformity.

Three years later, Fan and co-authors conducted a prospective cohort study analyzing the influence of PSSE physiotherapy on different types of (thoracic or lumbar) scoliosis. The authors used the BSPTS method (*n* = 40) and tracked the results for 1.5 years after 6 months of treatment. They observed no difference between thoracic and lumbar scoliosis [64], which suggests that the type of spinal deformity does not have a significant influence on the treatment of AIS with PSSE physiotherapy. Progression of deformity was not observed in either the lumbar or the thoracic scoliosis group, as the Cobb angle regressed in 20% of cases (*n* = 8) and stabilized in 80%. In 65% of patients, skeletal maturity (Risser 5) was reached within 2 years after the initiation of the treatment. This proves that BSPTS is an effective treatment intervention for mild-to-moderate deformities [64]. However, due to the small sample size, detailed studies of the methodologies should be carried out to confirm the results.

To investigate the effectiveness of the FED methodology, Nisser and co-authors conducted a systematic analysis of one randomized controlled clinical trial [49] and eight retrospective cohort studies. In the majority of studies, FED intervention was found to result in a statistically significant positive change in posture parameters and regression or stabilization of the Cobb angle. However, due to the limitations of the studies, the use of different protocols, the lack of quality studies, and the complexity of the therapy, the authors were unable to draw conclusions about the effectiveness of FED. Trzcińska and co-authors further explored this methodology in a randomized controlled clinical trial in 2020. The authors found that FED and FITS methodologies caused a significant reduction in the Cobb angle, with FED being advantageous over FITS. However, the study lasted only 3 weeks, and the sample included only 60 patients [25]. Thus, although studies on the effectiveness of the FED methodology show clinically significant changes, the existing evidence lacks scientific validity.

Similar limitations have been observed for the studies on the effectiveness of other PSSE physiotherapy methodologies, as the scientific literature on the DoboMed, FITS, and Lyon methodologies is lacking. The DoboMed methodology has not been widely studied over the past decade, and there is only one retrospective study [65] providing low-quality evidence that the methodology helps to stabilize the progression of scoliosis. Białek proved that the FITS method has a stabilizing effect on the progression of scoliosis, but the author examined juvenile and adolescent scoliosis together [66]. Mamyama and co-authors investigated the effect of the Side Shift technique on scoliotic patients after skeletal maturity (Risser 5) and noted no change in deformation for over 4.2 years in 44 out of the 69 patients [67]. In the remaining patients, the deformation angle regressed to 1.2°, but the change was statistically insignificant. According to Berdishevsky and co-authors, the effectiveness of the Lyon methodology has not been scientifically proven in the cases of mild scoliosis (Cobb < 20°) [15]. The authors stated that when the Cobb angle is greater than 20°, the effectiveness of the technique relies primarily on bracing. No specific controlled clinical trials investigating the effectiveness of the Lyon methodology can be found in the databases we accessed. Thus, the reviewed scientific literature indicates that the Cobb angle of scoliosis can be stabilized or even reduced by applying Schroth, SEAS, BSPTS, and FED methodologies, but the scientific validity of the FITS, Lyon, DoboMed, and Side Shift methodologies is questionable.

#### 3.1.2. Effects of PSSE Physiotherapy on the Angle of Trunk Rotation

In the meta-analysis of Anwer and co-authors [29], two [43,50] randomized controlled clinical trials were reviewed to evaluate the effects of physical exercise on ATR. Both studies were rated above 6 on the PEDro scale and provided moderate-quality evidence that exercise interventions caused a statistically significant reduction in ATR. The level of heterogeneity of the studies was found to be low (*I*^2^ = 1.49%).

Thompson and co-authors reviewed three randomized controlled clinical trials to investigate the effects of PSSE physiotherapy methodologies on ATR. Two [43,50] of those trials were also included in the meta-analysis of Anwer and co-authors. Quality assessment revealed that the third [68] study was characterized by a high risk of bias. Based on this study, the results of Anwer and co-authors were supplemented by a finding that patients who received a PSSE physiotherapy intervention showed a 4.4° mean decrease in lumbar ATR (95% CI [–7.6; –1.2]), but no statistically significant change in thoracic ATR was detected [59]. 

Among the most recent controlled clinical trials (Table 3), six recorded a change in ATR [37,38,41,43,45,46]. In two trials, in which bracing was used in the control group, no statistically significant changes in ATR were observed [41,46]. In one study [45], no statistically significant change in ATR was observed between the intervention group and the observation control group, whereas in a short-term (2 week) study on Schroth conducted with a small sample (*n* = 50) [37], a significant change in ATR was observed (from 6.04° to 5.32°, *p* < 0.01). However, the difference between the groups was insignificant. Kocaman and co-authors conducted a short-term (10 week) study on a small sample receiving Schroth intervention (*n* = 28) and observed a significant (*p* < 0.001) change in ATR in the thoracic scoliosis group, but an insignificant change in the lumbar scoliosis group (*p* > 0.05) [38]. A lumbar muscle stabilization program was used in the control group. In another study (*n* = 45), Kuru et al. found a –4.23° (*p* < 0.001) change in ATR in the specialist-supervised Schroth group at week 24 compared with the group subjected to a home exercise program (Schroth) and the control group [43]. Thus, the most recent controlled clinical trials indicate that only the Schroth methodology significantly reduces ATR. However, it must be noted that these data were obtained only from short-term studies and using a small sample.

#### 3.1.3. Effects of PSSE Physiotherapy on the Quality of Life

Anwer and co-authors [29] reviewed three studies in their meta-analysis, two of which were randomized controlled clinical trials [43,69], with an aim of investigating the impact of physical exercise interventions on the quality of life. All included studies were rated above 6 on the PEDro scale, and statistical analysis revealed that exercises improved the quality of life of patients. A high degree of homogeneity was found (*I*^2^ = 0) [29].

Burger and co-authors [32] analyzed two randomized controlled clinical studies [43,70], one of which was also reviewed by Anwer et al. [43], to determine the effects of PSSE physiotherapy on quality of life. Both studies (in which the Schroth intervention was applied) were rated above 6 on the PEDro scale. A meta-analysis was performed separately for 12 week and 24 week programs. A statistically significant improvement in quality of life was found in the 12 week group (mean difference = 0.72, 95% CI [0.25; 1.18]) and the 24 week group (mean difference = 0.83, 95% CI [0.37; 1.29]) receiving PSSE physiotherapy interventions [32].

The study by Thompson and co-authors [59] included two randomized controlled clinical trials [68,70], one of which was also reviewed by Burger and co-authors [70]. The newly included study had a high risk of bias, providing low-quality evidence that PSSE physiotherapy increases quality of life (standard mean difference = 0.25, 95% CI [0.21; 0.29]). The data were characterized by high homogeneity (*I*^2^ = 0%) [54].

Among the most recent controlled clinical trials (Table 3), seven [37,38,41,43,46,47,56] recorded the effect of PSSE physiotherapy on quality of life, although no significant difference in this parameter was observed in three of them [43,46,56]. A short-term (2 week) study used the Schroth methodology in a sample of *n* = 50 [37]. It was observed that SRS-22 (the Quality of Life questionnaire of the Scoliosis Research Society) data were slightly more favorable in the Schroth group (only in the treatment satisfaction domain) (4.42 ± 0.66 versus 4.18 ± 0.92). In a 10 week study (*n* = 28), Kocaman and co-authors noted that the Schroth intervention was superior to trunk endurance exercises, but their analysis did not evaluate individual domains (Schroth group: 4.56, control group: 4.3, *p* < 0.05) [38]. Another study (*n* = 48) found that Schroth was better at improving the domain of functional activity (4.8 versus 4.6). The study was performed for 18.1 ± 6.2 months, and bracing was used in the control group [41]. In another study, the SEAS methodology caused a significant improvement in the domain of functional activity (4.9 versus 4.7) and in the domain of mental health [47]. The study (*n* = 53) lasted 1 year, and bracing without physical exercise intervention was used in the control group. This suggests that PSSE physiotherapy can cause a significant improvement in the domain of functional activity (two long-term studies on Schroth and SEAS) and in the domain of mental health (a long-term study on SEAS). Two other short-term studies demonstrated that Schroth exercises were advantageous over the control intervention in improving the overall SRS-22 score or the treatment satisfaction domain alone.

The scientific publications on the effectiveness of PSSE physiotherapy methodologies reviewed in this work revealed that only Schroth and SEAS methodologies have been widely studied so far, whereas studies on the effectiveness of BSPTS, FED, FITS, DoboMed, and Side Shift methodologies are lacking. The reviewed studies are diverse, with meta-analyses and systematic analyses highlighting the heterogeneity of study protocols, which complicates the systematization of high-quality evidence for justifying the effectiveness of PSSE physiotherapy. Nevertheless, data from the studies indicate that the Schroth methodology alone can cause a statistically significant change in ATR, but these studies were based on a small sample and conducted for a short duration. Both Schroth and SEAS are statistically effective in terms of improving the quality of life. With respect to the Cobb angle (the main indicator of the effectiveness of the scoliosis treatment), the available data suggest that this parameter can be stabilized and/or even reduced by Schroth, SEAS, BSPTS, and FED techniques. Only two studies have compared the effects of the above methodologies; of these, one study provided statistically significant evidence of Schroth’s advantage over SEAS, and the other proved that FED is superior to FITS. In both these studies, the effectiveness of the methodologies was examined only based on the changes in the Cobb angle. Due to the limitations of these two studies, detailed research is needed to support their conclusions. However, it can be stated that PSSE physiotherapy is beneficial for the management of mild-to-moderate scoliosis, and a positive effect can also be expected in cases with severe or very severe deformities.

## 4. Discussion

Adolescent idiopathic scoliosis is a complex orthopedic pathology, which should be treated during the growth period using a targeted treatment plan to achieve optimal therapeutic outcomes. Conservative treatment is still preferred for scoliosis management, which aims to avoid or delay the need for surgical treatment as much as possible [8]. Conservative scoliosis treatment helps to reduce or stabilize the deformity and improve the esthetics of the deformation. Traditional bracing has been beneficial in the management of AIS, but growing evidence suggests that PSSE physiotherapy, based on self-correction, stabilization of the corrected posture, and training of daily living activities, is a more effective intervention. This PSSE physiotherapy is usually performed by following a specific methodology. A review of the scientific literature revealed some similarities between the PSSE physiotherapy methodologies. In the Lyon and FED methods, PSSE physiotherapy is used as an adjacent therapy; bracing is the main treatment in the Lyon method [15,71], while the FED method involves mechanotherapy using a specific apparatus [49]. The SEAS methodology, which does not emphasize bracing, has been developed based on the Lyon method, and was modified over time according to scientific discoveries [15,72]. The DoboMed method focuses on exercises to increase chest kyphosis, which is typical of the Lyon method [73]. This method uses a “derotational” breathing that was first applied to Schroth, the most widely studied among the PSSE physiotherapy techniques. The Schroth technique is primarily based on isometric muscle contraction exercises aiming to rotate, lengthen, and stabilize the spine [15,19]. The BSPTS method was formed following the principles of the Schroth methodology, and includes cognitive, sensory, and kinesthetic training [14]. The Side Shift technique is based on special corrective trunk side shift exercises [26], and the FITS methodology was developed in accordance with the principles of various PSSE physiotherapy schools [73]. All the mentioned methodologies more or less focus on applying corrective exercises in three planes, developing spinal stability and balance, breathing exercises, and posture awareness.

The effectiveness and scientific validity of PSSE physiotherapy depends on the applied methodology. Over the last decade, scientific publications have focused mainly on Schroth and SEAS, but less on FED and BSPTS, and very little attention has been paid to the DoboMed, Side Shift, and FITS methodologies. Some of the reviewed publications did not differentiate between the used methodologies, and regardless of the methodology used, the results were presented in the context of PSSE physiotherapy [34,59]. A meta-analysis by Thompson and co-authors revealed that PSSE physiotherapy caused a statistically significant reduction in the Cobb angle; however, many of the studies reviewed by these authors had a high risk of bias and followed a low-quality protocol [59]. The meta-analyses performed by other authors to investigate the effects of PSSE physiotherapy failed to produce reliable results due to the high heterogeneity of the examined studies [32,34,48]. Some studies considered interventions, such as the trunk stabilization exercise program, as PSSE physiotherapy [68], while some used trunk stabilization exercises in the control group [38]. Thus, it is appropriate to analyze the effectiveness of PSSE physiotherapy methodologies separately in order to obtain evidence of higher quality.

The effectiveness of PSSE physiotherapy methodologies has traditionally been measured based on the following two main indicators: Cobb angle and ATR. Park and co-authors observed a higher effect for the Schroth methodology when the therapy lasted for more than 1 month [48], whereas Burger and co-authors noted higher effectiveness for this technique in short-term studies [32]. Considering that the scoliosis treatment plan lasts on average 2–3 years, it is important to evaluate the effectiveness of the methodologies in the long run. Long-term studies have revealed the statistically significant effect of the SEAS, BSPTS, and Schroth methodologies in the treatment of AIS. A study by Negrini and co-authors [45] demonstrated that the Cobb angle increased by 1.70° ± 7.24° in the experimental group during 25 months of treatment (*p* < 0.05 between groups). In a study on BSPTS, Zapata and co-authors noted that the effectiveness of the methodology was evident after 1 year, but after 6 months, the difference in Cobb angle between groups was insignificant [56].

A study on the effectiveness of Schroth showed not only stabilization of scoliosis but also regression [44,45]. In an 18 month study, Cobb angle decreased in 17%, stabilized in 62%, and increased in 21% of participants in the Schroth group; however, the sample size of the study was small (*n* = 48) [41]. Shah and co-authors [39] compared Schroth and SEAS methodologies and found a statistically significant advantage of Schroth between groups (*p* < 0.001), but this study also used a small sample (*n* = 30), and lasted only 7 weeks. The finding of this study is in line with the observations of Burger and co-authors [32] that the effect of the Schroth methodology is often stronger in shorter-term studies. In any case, more detailed studies are needed to support the effectiveness of the Schroth, SEAS, and BSPTS methodologies, and the scientific validity of other methodologies in the reviewed publications is questionable.

Evidence in peer-reviewed publications is insufficient to assess the effect of PSSE physiotherapy methodologies on ATR and quality of life. A meta-analysis by Thompson et al. revealed that patients who received PSSE physiotherapy showed a mean reduction in ATR of 4.4° [59]. However, the studies included in their analysis had a high risk of bias. A review of recent clinical trials showed that the use of the Schroth methodology alone caused a significant reduction in ATR [38,43,49]. However, the analyzed data were obtained from a small sample and short-term studies. In other studies, no significant difference in ATR was observed between groups. Studies evaluating the effect of PSSE physiotherapy on quality of life showed that it had a positive impact on the domain of functional activity (two long-term studies on Schroth and SEAS [41,47]) and the domain of mental health (a long-term study on SEAS [47]). Two other short-term studies showed that Schroth was more effective than the control intervention in improving the overall SRS-22 score or the treatment satisfaction domain alone [37,38]. However, more detailed research is needed to confirm the correctness of these conclusions.

The comparison between different PSSE physiotherapy methodologies is limited in the reviewed publications. In one study, Shah and co-authors [39] found a statistically significant advantage of Schroth over SEAS, and in another study [49], Nisser and co-authors found that FED had an advantage over FITS. One review also investigated PSSE physiotherapy techniques with the aim of identifying the superior treatment [74]. According to the authors, the SEAS and DoboMed methodologies are inferior to Schroth and Side Shift. This was attributed to the development of the Schroth and Side Shift program for a specific deformity and type of scoliosis; however, such conclusions are not based on specific statistics. The authors cited three studies to support their argument [43,50,70], but none of the studies compare the methodologies with each other or examine them in parallel. In addition, although the SEAS methodology does not have a separate classification of spinal deformities, its protocol involves active self-correction and personalization of physical exercise for each patient [18]. As a result, the validity of the findings presented in the publication is questionable, and the evidence provided for the advantage of Schroth and the FED over other methodologies requires more investigation.

Most of the authors of the reviewed publications have stated that it is impossible to draw reliable conclusions about the effectiveness of PSSE in the treatment of AIS due to the low quality of the results, which is related to a small sample size, a short study duration, differences in the applied intervention, and variability in the control group (observation, bracing, exercises, and traditional physiotherapy). Moreover, a number of aspects vary between patients, including Risser sign, type of scoliosis, degree of deformity, bracing, intensity of therapy, and consistent execution of the program. Therapy may be individualized and, thus, differ between patients. It is also challenging to organize randomized clinical trials on this subject, as a lack of treatment in the control group raises ethical concerns. However, future research on conservative treatment of AIS should be based on long-term cohort studies. In addition, only two studies have so far compared PSSE physiotherapy interventions with each other. Therefore, it is important to compare the effectiveness of these methodologies.

The Schroth method has been the most studied in the reviewed publications and, according to recent studies, only this method allows for a statistically significant reduction in the angle of spinal axial rotation. However, Schroth, SEAS, and BSPTS methodologies are effective in inhibiting the progression of scoliosis. In any case, the available evidence is not strong enough, so longer-term and larger-sample studies are needed to obtain more reliable results.

## 5. Conclusions

Inappropriate treatment of adolescent idiopathic scoliosis can result in serious health problems. Conservative interventions are preferred for scoliosis management as they help to stabilize spine curvature and improve aesthetics. Bracing has traditionally been the mainstay of treatment, but growing evidence indicates that physiotherapeutic scoliosis-specific exercises (PSSE) may be more effective in managing adolescent idiopathic scoliosis. There are eight PSSE physiotherapy schools in Europe, as follows: Schroth, SEAS, BSPTS, FED, FITS, Lyon, Side Shift, and DoboMed. All of their methodologies are similar to each other and focus on applying corrective exercises in three planes, developing stability and balance, breathing exercises, and posture awareness.

There is still a lack of high-quality research supporting the effectiveness of PSSE physiotherapy in the treatment of AIS but, according to the existing evidence, PSSE physiotherapy helps to stabilize spinal deformity and improve quality of life. Schroth methodology is the most widely studied and has proven to be effective. However, both SEAS and BSPTS also can effectively stabilize or even reduce the Cobb angle of scoliosis. Data supporting the validity of other methodologies are very limited. Only the Schroth method has been shown to significantly reduce angle of trunk rotation, while SEAS and Schroth significantly improve the quality of life indicators. Nonetheless, the available evidence is insufficient to confirm the advantage of one specific physiotherapy technique over others, and more high-quality research is still needed. By bringing the existing knowledge together, the current review offers potential routes for further exploration and research in this field.

## Figures and Tables

**Table 1:** Physiotherapeutic scoliosis-specific exercises (PSSE) methods.

No.	Methodology	Country of Origin	Description
1.	Schroth	Germany	The Schroth methodology is one of the most widely used and researched in the scientific literature. Its success is credited to its proprietary Schroth rotational angular breathing (RAB) technique. It is a three-dimensional treatment for scoliosis with a focus on the pattern-specific postural correction according to the Schroth’s classification system of scoliosis. Mirror monitoring allows the patient to synchronize the corrective movements and postural perceptions, and to receive immediate visual feedback. The five principles of the Schroth method are auto-elongation (detorsion), deflection, derotation, rotational breathing, and stabilization. Since Schroth was created, various branches of the school emerged [15,19,20].
2.	Lyon	France	Physiotherapeutic treatment includes 3D mobilization of the spine, mobilization of the iliolumbar angle (lumbar scoliosis), patient education, and activities of daily living, including correction of the sitting position. The basis of the Lyon method is to avoid spinal extension during exercise and to enhance kyphosis of the thoracic region with lordosis of the lumbar spine as well as frontal plane correction, segmental mobilization, core stabilization, proprioception, balance, and stabilization [15].
3.	SEAS (Scientific Exercise Approach to Scoliosis)	Italy	The SEAS exercises are based on autocorrection and stabilization. The SEAS exercises have the following two main objectives, in order of importance: 1. The exercises aim to improve the main spinal function, i.e., spinal stability; 2. The exercises aim to improve eventual impairments that the initial evaluation may highlight (strength, muscular retraction, motor coordination, etc.) [18].
4.	BSPTS (Barcelona Scoliosis Physical Therapy School)	Spain	The BSPTS technique is based on the original Schroth method. The principles of correction follow the global postural alignment and are applied with high intensity forces created inside the body (‘from inside’) involving isometric tensions, expansions, and specific breathing. The BSPTS concept is based on four general principles, as follows: 3D postural correction, the expansion/contraction technique, stabilization by muscle tension, and integration [15].
5.	Side Shift	UK	The Side Shift method’s technique is based on intensive trunk-bending training. This in an active form of autocorrection, in which the patient is taught to shift the trunk sideways over the pelvis in the direction opposite to the convexity of the primary curvature [15,21].
6.	DoboMed	Poland	The DoboMed method focuses on deepening the thoracic kyphosis, carried on in closed kinematic chains, and developed on a symmetrically positioned pelvis and shoulder girdle, followed by active stabilization of the corrected position, and endured as postural habit. It also includes the rotational angular breathing exercise of Schroth [22].
7.	FITS (Functional Individual Therapy of Scoliosis)	Poland	The FITS methodology stands for Functional Independent Treatment for Scoliosis. It consists of two stages, as follows: the detection and elimination of myofascial restrictions, and the construction of a series of new corrective posture patterns in everyday activities [15,23].
8.	FED	Spain	The name of the method, FED, is an acronym of three words, namely F—fixation, E—elongation, and D—derotation. The FED methodology is described as a three-dimensional stabilization of the spine with its simultaneous extension and derotation. It uses a sophisticated mechanotherapy device for treatment, which enables corrective forces to act at the level of the scoliotic curve [15,24,25].

## Data Availability

Not applicable.
